# Differentiating between obstructive and non‐obstructive azoospermia: A machine learning‐based approach

**DOI:** 10.1002/bco2.493

**Published:** 2025-02-17

**Authors:** Abdolreza Haghpanah, Nazanin Ayareh, Ashkan Akbarzadeh, Dariush Irani, Fatemeh Hosseini, Farid Sabahi Moghadam, Mohammad Ali Sadighi Gilani, Iman Shamohammadi

**Affiliations:** ^1^ Department of Urology, School of Medicine Shiraz University of Medical Sciences Shiraz Iran; ^2^ Student Research Committee, School of Medicine Shiraz University of Medical Sciences Shiraz Iran; ^3^ Department of Compute Engineering, Faculty of Engineering, Mahshahr Branch Azad University Mahshahr Iran; ^4^ Department of Urology, Shariati Hospital, Faculty of Medicine Tehran University of Medical Sciences Tehran Iran; ^5^ Department of Andrology, Reproductive Biomedicine Research Center Royan Institute for Reproductive Biomedicine Tehran Iran

**Keywords:** azoospermia, machine learning, male infertility, obstructive

## Abstract

**Background:**

Infertility is a major global concern, with azoospermia, being the most severe form of male infertility. Distinguishing between obstructive azoospermia (OA) and non‐obstructive azoospermia (NOA) is crucial due to their differing treatment approaches. This study aimed to develop a machine learning model to predict azoospermia subtypes using clinical, ultrasonographic, semen and hormonal analysis data.

**Methods:**

This retrospective study included all subjects diagnosed with azoospermia. All patients were evaluated by at least one urologist, had their semen sample assessed on at least two different occasions for diagnosis and underwent a testicular biopsy to determine the type of azoospermia, categorized into OA and NOA. Clinical factors, hormonal levels, semen parameters and testicular features were compared between the OA and NOA groups. Three machine learning models, including logistic regression, support vector machine and random forest, were evaluated for their accuracy in differentiating the two subtypes.

**Results:**

The study included a total of 427 patients with azoospermia, of which 326 had NOA and 101 had OA. The median age of the patients was 33.0 (IQR: 7.0) years. Our findings revealed that factors such as body mass index, testicular length, volume and longitudinal axis, semen parameters and hormonal levels differed significantly between the two groups. When these variables were input into the machine learning‐based models, logistic regression achieved the highest F1‐score and area under the curve value among the three models evaluated.

**Conclusions:**

This study underscores the potential of machine learning to differentiate between azoospermia subtypes using readily available clinical data. However, further research is required to validate and refine the model before it can be applied clinically.

## 1 INTRODUCTION

Infertility is a significant medical issue, affecting an estimated 186 million people globally.^1^ Male infertility accounts for approximately 29% of all infertility cases.^2^ One cause of male infertility is azoospermia, defined as the absence of sperm in semen. Azoospermia is diagnosed when, on two separate occasions, a sperm sample shows no sperm upon examination under a high‐powered microscope after centrifugation.^3^ This condition impacts around 1% of the general male population and 10% of men who are infertile.^4^, ^5^, ^6^

Azoospermia is categorized into two types: obstructive azoospermia (OA) and non‐obstructive azoospermia (NOA), with OA representing approximately 40% and NOA about 60% of cases.^7^, ^8^, ^9^ Differentiating between these two subtypes is vital for patient management. Some prior studies have employed follicle‐stimulating hormone (FSH) levels and testicular size cut‐offs to differentiate between OA and NOA with notable sensitivity and specificity.^4^, ^10^, ^11^ To improve the distinction between these subtypes, we aimed to conduct a study using more precise models. In this study, we sought to develop a machine learning‐based model that predicts OA from NOA by leveraging characteristics that affect the quantity and quality of men's sperm. To the best of our knowledge, this is the first instance of machine learning‐based modelling being applied to differentiate between OA and NOA in the literature.

## 2 METHODS AND MATERIALS

### 2.1 Study design and patients

This retrospective study was conducted at the Royan Research Institute in Tehran, Iran, and two hospitals affiliated with Shiraz University of Medical Sciences in Shiraz, Iran. These institutions provide infertility treatments to patients in Iran and neighbouring countries. The data for patients diagnosed with azoospermia were collected from the Royan Research Institute from December 2016 to September 2020 and from Shiraz University of Medical Sciences from December 2016 to February 2022. The current study has received approval from the Ethics Committee of Shiraz University of Medical Sciences (IR.SUMS.MED.REC.1402.249).

The study encompassed all individuals diagnosed with azoospermia. Each patient was examined by at least one urologist, had their semen sample analysed on at least two separate occasions for diagnostic purposes and underwent a testicular biopsy to ascertain the type of azoospermia, which was categorized into OA and NOA.

### 2.2 Data gathering

The recorded information included as follows:

- Demographic information: age and body mass index (BMI)
- Past medical history: genitourinary tract infection, epididymo‐orchitis, undescended testis and orchiopexy, herniorrhaphy, varicocelectomy and vasectomy
- Physical examination findings: testicular volume and length
- Ultrasonography results: testicular longitudinal axis
- Semen analysis: semen volume, pH and fructose content
- Serum hormonal analysis: luteinizing hormone (LH), FSH and testosterone
- Genetic studies: karyotype and azoospermia factor

### 2.3 Statistical analysis

The collected data were analysed using the Statistical Package for the Social Sciences (IBM SPSS Statistics for Windows, Version 26.0, Armonk, NY: IBM Corp). The Shapiro–Wilk test was utilized to assess the distribution of continuous variables. Data are presented as mean ± standard deviation (SD) or median (interquartile range [IQR]). Univariate analysis included the Student's *t*‐test and Mann–Whitney *U* test for parametric and non‐parametric continuous variables, respectively, and the chi‐square and Fisher's exact tests for categorical variables, as appropriate. Outcomes with a *p*‐value less than 0.05 were considered statistically significant.

### 2.4 Machine learning modelling

Our primary objective was to develop a classification model that could categorize a patient with azoospermia as either OA or NOA, relying solely on the patient's clinical and ultrasonographic data. Other machine learning model families, such as deep learning, were deemed unsuitable for this study due to the relatively small size of our dataset. The model was programmed in Python 3.10.9, utilizing the Pandas and Scikit‐learn libraries. Three models—the support vector classifier (SVC) (gamma = ‘auto’, C = 1, kernel = ‘linear’), random forest classifier, and logistic regression—were selected for testing because they are renowned for their effectiveness with tabular data. To assess and compare the accuracy of the various classifiers, we employed the train‐test validation method. Specifically, patients were randomly allocated to either the training set or the test set. Each classification model was trained on the training set and then evaluated on the test set. A test size comprising 25% of the dataset was selected.

The GridSearchCV tool was utilized to conduct fivefold cross‐validation for each model during the hyperparameter optimization process. This approach facilitated the evaluation of model performance and the fine‐tuning of hyperparameters through iterative folding.

#### 2.4.1 Pre‐processing

Values that were not statistically significant (*p*‐value greater than or equal to 0.05) were excluded. Additionally, karyotype and vasectomy data were omitted. All five cases of vasectomy in the dataset were categorized as obstructive, which created an imbalance. Similarly, all abnormal karyotype cases in the dataset were labelled as non‐obstructive, further exacerbating the data imbalance. Such imbalances could lead the model to overfit, meaning it might learn to predict the majority class while neglecting the accurate prediction of the minority class. By removing these two variables from the training data, we achieved a more balanced dataset, which diminished the likelihood of overfitting and enhanced the model's ability to generalize. There were no instances of missing data.

#### 2.4.2 Performance evaluation

For the performance evaluation of the models, we calculated the area under the curve (AUC) of the receiver operating characteristics (ROC), F1‐score, accuracy, precision and recall. The model that achieved the highest F1‐score was selected as the superior model for each endpoint in the study. Additionally, it is generally recognized that an AUC of 0.5 signifies no ability to discriminate, an AUC between 0.7 and 0.8 is deemed acceptable, an AUC between 0.8 and 0.9 is considered excellent, and an AUC exceeding 0.9 is regarded as exceptional. The calculations for these metrics were conducted as follows:

$$\text{Accuracy}=\frac{\text{True positive}+\text{True negative}}{\text{True positive}+\text{True negative}+\text{False positive}+\text{False negative}}$$

$$\text{Precision}=\frac{\text{True positive}}{\text{True positive}+\text{False positive}}$$

$$\text{Recall}=\frac{\text{True positive}}{\text{True positive}+\text{False negative}}$$

$$F1-\text{score}=\frac{2\times \text{Precision}\times \text{Recall}}{\text{True positive}+\text{False positive}}$$

## 3 RESULTS

In a study of 427 patients with azoospermia, 326 had NOA and 101 had OA. The median age of the patients was 33.0 (IQR: 7.0) years.

### 3.1 Univariable analysis

The data of patients, categorized by the type of azoospermia, are presented in Table 1. The BMI was marginally lower in patients with NOA compared to those with OA (*p*‐value: 0.049). Additionally, chromosomal defects were observed exclusively in patients with NOA, while vasectomy was found only in patients with OA. A total of 65 patients in the NOA group (19.9%) had an abnormal karyotype, all of which were diagnosed with Klinefelter syndrome. Among the patients in the OA group, five patients (5.0%) had a history of vasectomy. The history of varicocelectomy, urinary tract infections, epididymo‐orchitis, hernia and undescended testis did not show statistical differences between the two types of azoospermia (*p*‐values >0.05). The testes of patients with OA were significantly larger in volume and length upon physical examination (both *p*‐values <0.001). The testicular longitudinal axis was also greater in the OA group (*p*‐value <0.001). In terms of semen analysis, semen volume, fructose and pH were lower in the OA group compared to the NOA group (all *p*‐values <0.001). Furthermore, patients in the NOA group exhibited higher levels of FSH and LH and lower levels of testosterone (all *p*‐values <0.001).

**TABLE 1:** Univariable analysis of patients' information.

Variables		Total (*n* = 427)	OA (*n* = 101)	NOA (*n* = 326)	*p*‐Value
Demographic information	Age, median (IQR) (years)	33.0 [7.0]	33.0 [6.5]	33.0 [7.0]	0.434
	BMI, median (IQR) (kg/m^2^)	26.0 [5.3]	26.6 [4.9]	25.7 [5.7]	**0.049**
Past history	Urinary tract infection, *n* (%)	4 (0.9%)	0 (0.0%)	4 (1.2%)	0.577
	Epididymo‐orchitis, *n* (%)	8 (1.9%)	4 (4.0%)	4 (1.2%)	0.094
	Undescended testis, *n* (%)	45 (10.5%)	13 (12.9%)	32 (9.8%)	0.382
	Hernia, *n* (%)	39 (9.1%)	12 (11.9%)	27 (8.3%)	0.273
	Varicocelectomy, *n* (%)	68 (15.9%)	17 (16.8%)	51 (15.6%)	0.776
	Vasectomy, *n* (%)	5 (1.2%)	5 (5.0%)	0 (0.0%)	**0.001**
Physical examination	Testis volume, median (IQR) (mL)	12.0 [14.0]	20.0 [5.0]	10.0 [11.0]	**<0.001**
	Testis length, median (IQR) (cm)	3.0 [2.0]	4.0 [0.0]	3.0 [2.0]	**<0.001**
Ultrasonography	Testis longitudinal axis, median (IQR) (mm)	37.0 [11.0]	40.0 [3.0]	34.0 [13.0]	**<0.001**
Semen analysis	Semen volume, median (IQR) (mL)	2.4 [2.4]	1.0 [1.5]	2.9 [2.0]	**<0.001**
	Semen pH, median (IQR)	7.8 [0.3]	7.0 [0.7]	7.8 [0.1]	**<0.001**
	Semen fructose, median (IQR) (mg/dL)	180.0 [150.0]	0.0 [120.0]	190.0 [120.0]	**<0.001**
Hormonal analysis	FSH, median (IQR) (mIU/mL)	11.3 [19.5]	3.8 [2.3]	15.9 [20.9]	**<0.001**
	LH, median (IQR) (mIU/mL)	7.1 [7.9]	4.1 [2.4]	8.8 [9.7]	**<0.001**
	Testosterone, median (IQR) (ng/mL)	3.4 [2.1]	3.8 [2.0]	3.1 [2.1]	**<0.001**
Genetic study	Abnormal karyotype, *n* (%)	65 (15.2%)	0 (0.0%)	65 (19.9%)	**0.007**
	Azoospermia factor, *n* (%)	5 (1.2%)	0 (0.0%)	5 (1.5%)	1.000

*Note*: Bold *p*‐values indicate statistical significance.

Abbreviations: BMI, body mass index; FSH, follicle‐stimulating hormone; IQR, interquartile range; LH, luteinizing hormone; NOA, non‐obstructive azoospermia; OA, obstructive azoospermia; SD, standard deviation.

### 3.2 Machine learning

**FIGURE 1:**
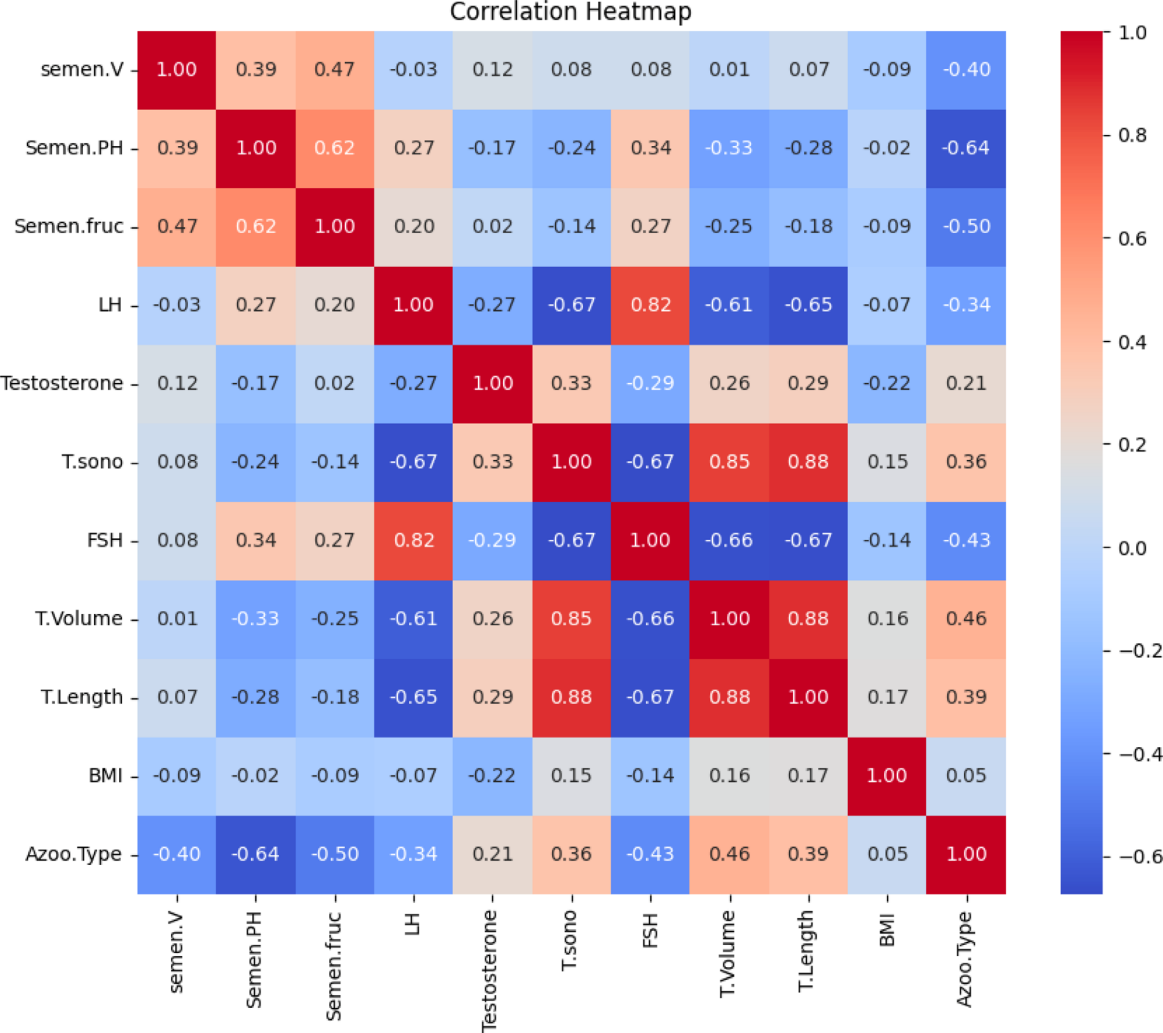
Correlation heat map of variables included in the machine learning‐based modelling; Azoo, Type of azoospermia; BMI, body mass index; FSH, follicle‐stimulating hormone; LH, luteinizing hormone; semen.fruc, semen fructose; semenPH, semen pH; semen. V, semen volume; T. Length, testis length; T.sono, testis longitudinal axis in ultrasonography; T. Volume, testis volume.

**FIGURE 2:**
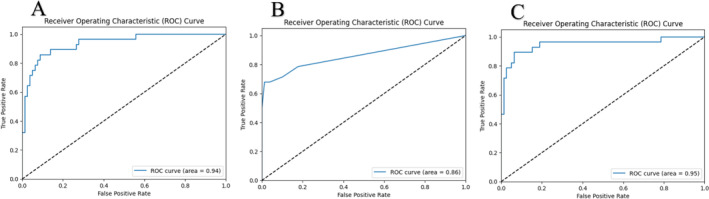
Area under the curve (AUC) of receiver operating characteristics (ROC) (A) support vector classifier, (B) random forest, and (C) logistic regression.

The machine learning‐based modelling included the following variables: BMI, testicular volume and length, testicular longitudinal axis, semen volume, pH and fructose, as well as FSH, LH and testosterone levels. Figure 1 illustrates the correlation heat map of these variables. The accuracy, F1‐score, recall and precision of the models are detailed in Table 2. Additionally, the ROC for all models is depicted in Figure 2. The logistic regression model achieved the highest AUC and F1‐score, indicating its superior performance in predicting the type of azoospermia (AUC: 0.95, F1‐score: 0.866). The model can be accessed on GitHub (https://github.com/FaridMoghadam/Predicting-Azoospermia-in-Male).

**TABLE 2:** Accuracy, F1‐score, recall, precision and AUC of the models.

Model	Accuracy	F1‐score	Recall	Precision	AUC
SVC	0.888	0.839	0.809	0.893	0.94
Random forest	0.888	0.843	0.820	0.879	0.86
Logistic regression	0.907	0.866	0.833	0.923	0.95

Abbreviations: AUC, area under the curve; SVC, support vector classifier.

## 4 DISCUSSION

Infertility has become an increasing global concern. Azoospermia, which is defined as the absence of sperm in semen fluid, is considered the most severe manifestation of testicular failure.^12^, ^13^ It is vital to have an error‐proof method of distinguishing the two different types of azoospermia in the early phases, due to their difference in treatment and prognosis.^8^ In OA, the goal of treatment is to correct the obstruction via reconstructive surgery. Otherwise, patients undergo sperm aspiration through testicular sperm extraction (TESE) for use in in vitro fertilization/intracytoplasmic sperm injection (IVF/ICSI). This contrasts with patients with NOA, who do not benefit from reconstructive surgery but should proceed to mTESE for surgical sperm retrieval.^14^ Hence, in our study, we developed a model that uses clinical factors, ultrasonographic findings and semen and hormonal analysis to predict the subtype of azoospermia. We found that logistic regression had the best performance. This model is accessible on GitHub (https://github.com/FaridMoghadam/Predicting-Azoospermia-in-Male). By further developing this model and making it applicable in diagnostic settings, we hope to create a tool that will further facilitate the diagnostic and treatment process for azoospermia.

As stated above, due to the differing treatment pathways, it is crucial to devise a reliable and efficient method for differentiating the two types of azoospermia. Among the diagnostic factors mentioned, testis biopsy and genetic studies appear to have the lowest margin of error and may be used as the standard tests in azoospermia patients. Despite this, biopsy procedures are invasive and carry many complications such as bleeding, hematoma, infection and, rarely, localized injury to the vascular supply. Often, patients are required to repeat the biopsy procedure.^15^, ^16^, ^17^ On the other hand, genetic studies are not accessible or affordable for everyone. Moreover, such diagnostic tests are usually time‐consuming and delay the onset of treatment. Therefore, there seems to be a need for a highly predictable, affordable and accessible method to differentiate OA from NOA.

We evaluated three machine learning models using clinical factors, ultrasonographic findings and semen and hormonal analysis. Among them, logistic regression emerged as the most accurate in distinguishing between the two types of azoospermia. The logistic regression model exhibited the highest AUC, indicating its proficiency in identifying the underlying causes of azoospermia. Logistic regression has also demonstrated its utility in various other infertility studies, suggesting its potential as a robust tool for predicting, diagnosing and differentiating azoospermia and infertility in general.^18^, ^19^, ^20^

### 4.1 Demographic information

The impact of age on male infertility has become increasingly significant in recent years, as more parents opt to have children later in life.^21^ In our study, which assessed the relationship between age and the type of azoospermia, no significant difference was observed between the two groups. This suggests that age may not be a determining factor in differentiating between OA and NOA.

It is well‐established that overweight men have a higher risk of developing azoospermia.^22^, ^23^ Furthermore, our research indicated that the median BMI was marginally higher in individuals with OA, but the results were not significant. Also, our research indicated that the median BMI was higher than normal range in both OA and NOA group. Supporting this, another study discusses the relationship between obesity and male infertility, particularly focusing on sperm quality and function,^24^ thereby affirming the significant role of BMI in both NOA and OA.

### 4.2 Past medical history

It is well‐documented that a history of urinary tract infections and epididymo‐orchitis is linked to male infertility, particularly OA.^25^, ^26^ However, our study did not reveal any significant correlation between these factors and OA, nor were they useful in distinguishing between the two types of azoospermia. This may be attributed to the very low number of individuals in our study who reported a history of urinary infections.

Cryptorchidism, especially when bilateral, is known to reduce spermatogenesis and consequently lead to infertility.^27^, ^28^ Although it is more common among NOA patients,^29^ our study indicated that it does not aid in differentiating between NOA and OA. The two most likely causes of OA in these patients are (1) the association of seminal duct anomalies with undescended testes and (2) obstruction caused by iatrogenic vas deferens transection or manipulation of the spermatic cord.^30^

Furthermore, factors such as a history of varicocele and varicocelectomy have been established as associated with infertility and are considered contributing factors to NOA.^14^, ^31^ While these factors are useful in diagnosing infertility, our study found that they do not assist in differentiating OA from NOA in patients.

### 4.3 Physical examination and ultrasonography

It is well‐established that testis length, as measured by physical examination or ultrasonography, is typically reduced in NOA, while it remains within the normal range in OA. Our study confirmed this finding and aligns with other research, such as the study by Huang et al.,^4^ which indicated that patients with NOA often have smaller bilateral testes. However, it should be noted that male patients with maturation arrest may exhibit normal testis size, a factor not assessed in our study.^32^ Furthermore, another study found that patients with NOA generally had a testicular long axis of 4.6 cm or less, whereas those with OA had a testicular long axis greater than 4.6 cm.^10^ Additionally, our study demonstrated that the average testis volume is typically lower in patients with NOA compared to those with OA.^33^

### 4.4 Semen analysis

Semen analysis can also be helpful in identifying OA patients, who are expected to have lower semen volume, lower fructose levels and lower pH.^25^, ^34^ The data gathered in our study showed the same results when comparing the two groups.

### 4.5 Hormonal analysis

An additional factor that helps distinguish OA from NOA is the levels of FSH, LH and testosterone. In the study by Schoor et al.,^10^ mean serum FSH and LH levels in NOA were significantly higher than those in OA. Similarly, the study by Huang et al.,^4^ showed higher levels of FSH and LH and lower levels of testosterone in NOA. These findings align with our investigation. Previous studies have suggested the use of a combination of serum FSH and testis size to accurately differentiate between OA and NOA.^4^, ^10^, ^11^ Although these studies utilized FSH and testis size, they had different cut‐off values. The study by Schoor et al.^10^ suggested that men with an FSH level of 7.6 mIU/mL or greater, or a testicular long axis of 4.6 cm or less, might be considered to have NOA. These cut‐offs were an FSH level of 9.2 mIU/mL and a right testis size of 1.5 cm in the study by Huang et al.^4^ for Taiwanese patients and an FSH level of 8.9 mIU/mL and a testicular long axis of 3.9 cm for the Iranian population.^11^ In the present study, we employed machine learning to minimize errors and maximize accuracy and prediction in patients with azoospermia. We discovered that logistic regression had the best performance, with an AUC of 0.95.

### 4.6 Genetic study

In some cases, NOA may be linked to genetic defects. Chromosomal abnormalities are observed in about 6% of men who suffer from infertility, with the highest risk in NOA.^14^, ^35^ In clinical practice, karyotyping and DNA extraction followed by multiplex polymerase chain reaction (PCR) are commonly employed to detect azoospermia factor microdeletions on the Y chromosome in patients with azoospermia.^36^, ^37^ Despite advancements in genetics and its significant role in evaluating patients with NOA, these tests are costly and, thus, not feasible in some clinical settings.

### 4.7 Limitations

This study was not without limitations. First, we did not assess the different types of NOA separately. Differences in hormone levels have been observed in these subtypes, which we did not evaluate in this study. Second, since spermatogenesis is not uniform across the testis, a single testis biopsy cannot reveal the complete pattern of spermatogenesis and may sometimes lead to diagnostic errors. Additionally, this is a retrospective study, which carries the inherent limitations of such investigations. An important point to note is that the results of studies similar to ours can be influenced by the population on which the study is based. We utilized machine learning to maximize the accuracy of predictions and minimize errors, but as every population may yield different results, we encourage further studies in diverse populations to differentiate between OA and NOA.

## 5 CONCLUSIONS

Testicular biopsy and genetic studies have remained the gold standard for diagnosis due to their high accuracy, despite their limitations. This study highlights the potential use of machine learning models in differentiating between azoospermia subtypes using readily available clinical data. However, further research is necessary to validate and enhance the model before it can be applied clinically.

## AUTHOR CONTRIBUTIONS

*Study concept and design*: Abdolreza Haghpanah, Nazanin Ayareh, Mohammad Ali Sadighi Gilani and Iman Shamohammadi. *Data acquisition*: Ashkan Akbarzadeh and Iman Shamohammadi. *Data analysis*: Dariush Irani and Fatemeh Hosseini. *Drafting of manuscript*: Nazanin Ayareh and Farid Sabahi Moghadam. *Manuscript editing*: Abdolreza Haghpanah and Iman Shamohammadi.

## CONFLICT OF INTEREST STATEMENT

All authors declare no conflict of interest.

## Data Availability

All data generated or analysed during this study are included in this published article. Also, the machine learning code can be accessed on GitHub (https://github.com/FaridMoghadam/Predicting-Azoospermia-in-Male).
